# Prevalence of kidney dysfunction in diabetes mellitus and associated risk factors among productive age Indonesian

**DOI:** 10.1007/s40200-018-0338-6

**Published:** 2018-03-27

**Authors:** Laurentia Mihardja, Delima Delima, Roy G. A. Massie, Muhammad Karyana, Pringgodigdo Nugroho, Em Yunir

**Affiliations:** 1 National Institute of Health Research and Development, Ministry of Health, Percetakan Negara no 29, Jakarta, Indonesia; 2 Department of Internal Medicine, Medical Faculty, University of Indonesia, Jakarta, Indonesia

**Keywords:** Kidney dysfunction, Diabetes mellitus, Prevalence, Risk factors

## Abstract

**Background:**

The prevalence of diabetes mellitus is increasing in Indonesia due to population growth, urbanization, and lifestyle. Diabetes mellitus (DM) is the leading cause of chronic kidney disease that escalates mortality rate, but not all DM develop into chronic kidney disease.

**Aims:**

To estimate the prevalence of kidney dysfunction (KD) in DM and the associated dominant risk factors among productive age Indonesian based on the National Health Survey (*Riskesdas*) 2013.

**Methods:**

The statistical data consisted of 15,791 females and 10,349 males, aged 20 to 54, who lived in rural and urban areas. The data was obtained from National Institute of Health Research and Development (NIHRD), Ministry of Health. Data were collected from 33 provinces using cross sectional method. The variables data analyzed were sociodemographic, lifestyle, anthropometric, blood pressure, blood lipid, blood glucose, and creatinine. Kidney dysfunction was defined according to Chronic Kidney Disease Epidemiology Collaboration (CKD-EPI) equation. Multivariable logistic regression was used to analyze the dominant associated risk factors.

**Results:**

The prevalence of KD in DM was 4% (CI 95% 3.1–5.1) and only 0.6% had been diagnosed. Many associated risk factors could affect DM leading to KD such as age, sex, rural, economic status, sugary food/drinks, salty food, coffee, hypertension, hypercholesterolemia, low HDL, high LDL, and hypertriglyceridemia. The dominant associated risk factors were age, sex, economic status, sugary food/drinks, and low HDL.

**Conclusion:**

The prevalence of KD in DM among productive age Indonesian was 4% and only 0.6% had been diagnosed. Early detection of identification of KD in DM is needed in order to slow progression and complications. The dominant associated risk factors of KD in DM were age, sex, economic status, sugary food/drinks, and low HDL. Controlling of risk factors in DM should be done in order to prevent diabetic kidney disease.

## Background

The prevalence of diabetes mellitus (DM) is increasing in Indonesia due to population growth, urbanization, and lifestyle. In 2007 the prevalence of DM in urban Indonesia was 5.7%, [1, 2], and in 2013 the prevalence of DM in urban and rural area was 6.9% [3]. Diabetes mellitus is the most common cause of kidney failure, accounting for approximately 44% of new cases of chronic kidney disease (CKD) [4]. Chronic kidney disease is a public health problem as it increases the mortality risk for any cause [5]. In the United States, about 24 million people had DM, and nearly 180,000 people were living with kidney failure as a result of DM [6]. In Indonesia, Prodjosudjadi W. et al. (2009), who researched people at the age of 18–70 in 4 provincial capitals, found that the prevalence of CKD was 12.5% (using Cockcroft – Gault = CG), 8.6% (using Modification of Diet in Renal Disease = MDRD), or 7.5% (using Chinese MDRD) [7].

The costs of care for people with diabetes kidney disease (DKD) are very high [8]. Patients with diabetes mellitus associated with renal impairment had an increased mortality risk, especially a higher risk of cardiovascular (CV) death, when compared to other diabetic patients without renal impairment [9]. Based on the data of National Health and Nutrition Examination Survey from 1999 through 2006, Plantinga L.C. et al. (2010) found CKD prevalence was 39.6% in diagnosed diabetes mellitus (DDM) and 41.7% in undiagnosed diabetes mellitus (UDDM) [10] Mihardja l. et al. (2009), found in Indonesia that the prevalence of DDM was 1.5% and UDDM 4.2% [2].

Not everyone with DM develops kidney disease. Many factors can influence kidney disease development such as genetics, blood sugar, blood pressure, and smoking [11]. Identification of risk factors that can lead to CKD in DM are important, because some of the risk factors can be modified and can prevent to CKD.

The objective of this study was to estimate the prevalence of kidney dysfunction (KD) in diabetes mellitus (DM) and its associated dominant risk factors in productive age Indonesian based on the National Health Survey (*Riskesdas*) 2013. This study will provide inputs on preventing KD in DM to the stakeholders.

## Methods

### Sampling and collecting the data

This study utilized data from Indonesia National Health Survey (NHS) 2013 or in local term called *Riskesdas*. The data was obtained from National Institute of Health Research and Development (NIHRD), Ministry of Health. The Indonesia NHS used a four-stage stratified sampling method. The first strata utilized systematic sampling of group of census blocks in all districts. The second strata used a proportional to enrolment size design to identify two census blocks followed by systematic sampling of 25 census buildings. Finally, random sampling was done to choose one household within the census buildings. The total samples of selected households were 300,000 households from 12,000 census blocks (as samples representing the districts.) Blood samples were selected from 1000 census blocks that consisted of 25,000 households as subsamples by systematic sampling, representing urban and rural areas across Indonesia. Respondents whose blood was taken had to be 15 years old or older, were not severely ill, were not taking anticoagulant medicines, and did not suffer from bleeding disorders [3].

The statistical data in this study consisted of 15,791 females and 10,349 males (total 26,140 people), aged 20 to 54, whose blood were taken as samples and lived in rural and urban areas. Cross-sectional data were collected from 33 provinces. A structured questionnaire was designed for data collection. Weight was measured using a digital scale, and height was measured using a microtoise, a mechanical measuring tape. Waist circumference was measured using a centimeter scale. Blood pressure was measured using a digital blood pressure monitor. Blood glucose of fasting and 2 hours after loading 75 g anhydrite glucose were measured using a glucometer. Fasting lipid profiles (total cholesterol, LDL cholesterol, HDL cholesterol, triglycerides) and creatinine were measured using the automatic clinical chemistry analyzer in government laboratory clinic. Data were collected by trained local enumerators having at least a bachelor’s degree. The survey was conducted by National Institute of Health Research and Development (NIHRD.) The research protocol was approved by the Ethics Committee of the National Institute of Health Research and Development. All subjects gave their consent to take part in the survey after receiving both written and oral information about the purpose of the research [3, 12].

### Definitions

Kidney dysfunction (KD) was defined by estimating glomerulus filtration rate (GFR) <60 ml/min per 1.73 m^2^ using Chronic Kidney Disease Epidemiology Collaboration (CKD-EPI) equation. We could not use CKD term because labelling someone as having CKD required two samples of at least 90 days apart [13]. Although Indonesia is an Asian country and it is known there have been several coefficient correction of CKD EPI formula for eGFR in Asia (Japan, China, Thailand), but we determined KD based on the actual formula of CKD-EPI creatinine equation: 141 x min(SCr/κ,1)^α^ x max(SCr/κ,1)^-1.209^ × 0.993^Age^ x [1.018 if female] [× 1.159 if black]. This is due to the coefficients correction in Asian obtained differ from one another, resulting in different eGFR and there has not been a coefficient consensus for Asians [14, 15].

Diabetes mellitus was defined according to WHO 2006 criteria and *PERKENI* (The Indonesian Society of Endocrinology) 2011 guidelines. Diabetes mellitus was defined as fasting blood glucose level ≥ 126 mg/dL or random blood glucose ≥ 200 mg/dL with four classic symptoms (increased thirst, increased hunger, frequent urination, and weight loss), blood glucose of 2 hours after a glucose load ≥200 mg/dl, or the use of glucose-lowering drugs [16, 17].

Undiagnosed Diabetes Mellitus (UDDM) was given if the subjects didn’t know they were suffering from DM.

Diagnosed Diabetes Mellitus (DDM) was given for subjects who were previously diagnosed DM by the doctor.

Diabetes Mellitus consisted of UDDM and DDM.

Body Mass Index (BMI) was calculated as weight (kg) divided by the square of the height (m). Obesity was defined as BMI ≥ 25 kg/m^2^. Overweight was defined as BMI ≥ 23 - < 25. Normal was defined as BMI >18.5 - < 23, and thin was defined as BMI < 18.5 (Asia Pacific WHO criteria) [18].

Central obesity was defined as waist circumference ≥ 90 cm in man, and ≥ 80 cm in women [19].

Hypertension was defined as systolic blood pressure ≥ 140 mmHg and/or diastolic blood pressure ≥ 90 mmHg; or when subject was using antihypertensive medications [20].

Dyslipidemia was defined as total cholesterol ≥200 mg/dL and/or LDL cholesterol ≥150 mg/dL and/or HDL cholesterol < 40 mg/dL in men and < 50 mg/dL in women, and/or triglycerides ≥ 150 mg/dL [21].

### Statistical analysis

The variables analyzed were sociodemographic, lifestyle, anthropometric, blood pressure, blood lipid, blood glucose, and creatinine. Data analysis was performed using the SPSS 16.0 software for complex samples. Multivariable logistic regression was used to analyze the dominant of associated risk factors. To understand about associated dominant risk factors of kidney dysfunction in diabetes mellitus, only the data of undiagnosed diabetes mellitus (UDDM) was analyzed because people with diagnosed diabetes mellitus (DDM) might be influenced by intervention like diet, physical activity, or anti diabetes medicine.

## Results

The prevalence of diabetes mellitus among productive age Indonesian was 6.5% (CI 95% 6.1–7.0), while diagnosed DM was only 1.8% (CI 95% 1.6–2.1). The prevalence of kidney dysfunction was 1.7% (CI 95% 1.4–1.9), and diagnosed KD was only 0.3% (CI 95% 0.2–0.4). The prevalence of KD in DM was 4% (CI 95% 3.1–5.1), while diagnosed KD in DM was only 0.6% (CI 95% 0.3–0.9).

The data in Table 1 depicted the prevalence of KD in UDDM and in DM, which affected more males than females and increased by age. Prevalence of KD in DM was higher in urban than in rural area, but prevalence of KD in UDDM was higher in rural than urban. Those who graduated from junior/senior high school had higher prevalence of KD in UDDM compared to those who graduated from primary school or not graduated. Prevalence of KD in UDDM was higher in farmers/fishermen/laborers compared to the other groups. Prevalence of KD in UDDM and in DM was lower in high economic society compared to low and middle economic society. Prevalence of KD in UDDM was higher in people who liked to consume sugary food/drinks every day than in people who did not comsume them every day, as well as people who liked to eat salty food, but the prevalence was lower in people who liked to drink coffee every day than in those who did not (very rarely/never). Prevalence of KD in UDDM was higher in those who smoked every day/any current or former than who never smoked. Prevalence of KD in UDDM was lower among those who lacked physical activity compared to the group that did sufficient physical activity, and it was higher in hypertension group than in no hypertension group. Prevalence of KD in thin group was lower than in normal, overweight, or obese group. Prevalence of KD tended to increase in UDDM and DM with hypercholesterolemia, high LDL, hypertriglyceridemia, and low HDL.

**Table 1:** Prevalence of kidney dysfunction in UDDM and DM based on characteristics, habits and biologic variables in Indonesia

Variables	Kidney Dysfunction			
	UDDM (*N* = 1183)		DM (*N* = 1506) (UDDM + DDM)	
	%	95% CI	%	95% CI
Characteristics				
Age (years)				
20–34 35–44 45–54	0.4 2.6 4.1	0.1–2.7 1.6–4.5 2.7–6.0	0.3 2.6 5.8	0.1–2.4 1.6–4.2 4.2–7.8
Sex				
Male Female	5.0 2.4	2.9–8.5 1.6–3.4	5.8 3.3	3.7–8.9 2.5–4.5
Residence				
Urban Rural	2.2 3.7	1.1–4.3 2.7–5.0	4.2 3.6	2.8–6.3 2.7–4.9
Education level				
Graduated from primary school or not Graduated from Junior/Senior High School Graduated Academy /University	2.4 4.4 -	1.5–3.8 2.9–6.5 -	3.7 4.2 4.6	2.7–5.2 2.9–6.1 1.7–12.3
Occupation				
Jobless Employee/private business Farmers/Fishermen/Laborers	2.6 2.0 4.1	1.6–4.2 0.9–4.3 2.6–6.4	4.1 3.4 3.7	3.0–5.6 1.9–6.2 2.3–5.7
Economic Status				
Quintil-1. 2 (low economic) Quintil-3 (middle economic) Quintil-4.5 (high economic)	3.8 4.3 1.6	2.5–5.3 2.4–7.7 0.9–3.0	4.3 4.2 3.6	2.9–6.5 2.5–7.1 2.4–5.4
Habits				
Fruit Vegetables				
≥ 5 portions/day 1–4 portions/day Never	- 3.1 -	- 2.2–4.3 -	- 3.9 -	- 2.9–5.2 -
Sugary food/drinks				
Every day (> = 1×/day) Not every day(1-6×/week) Very rarely or never(<=3×/month or never)	4.3 2.1 -	3.1–5.9 1.0–4.6 -	4.2 3.0 5.0	2.9–6.1 1.7–5.2 3.1–7.9
Salty food				
Every day Not every day Very rarely or never	3.5 2.8 2.8	2.1–6.0 1.6–4.8 1.8–4.4	3.6 4.0 5.3	2.1–6.0 2.6–6.0 2.9–6.3
Coffee				
Every day Not every day Very rarely or never	1.9 2.6 3.7	1.0–3.6 1.4–5.0 2.4–5.7	2.6 2.1 5.2	1.5–4.5 1.1–4.0 3.8–7.
Smoking				
Every day/any current/former Never	3.3 2.9	1.5–7.0 2.1–4.0	4.1 3.9	2.3–7.2 3.0–5.2
Physical activity				
Sufficient Lacking	3.1 2.5	2.2–4.3 1.2–5.5	3.9 4.1	3.0–5.2 2.4–7.1
Biologic				
Hypertension				
Hypertension No hypertension	3.2 2.6	2.1–4.8 1.6–4.2	4.5 3.4	3.1–6.4 2.3–5.0
Body mass index (BMI)				
Thin Normal Overweight and obese	2.1 3.5 2.7	0.5–8.5 1.7–6.7 1.9–3.8	1.8 4.0 3.9	0.4–7.0 2.4–6.7 3.0–5.2
Obesity				
Central No central obesity	2.5 3.3	1.8–3.6 1.9–5.6	3.7 4.0	2.8–5.0 2.6–6.1
Hypercholesterolemia				
total cholesterol ≥200 mg/dl < 200 mg/dl	3.6 2.5	2.4–5.2 1.5–4.2	4.5 2.4	3.2–6.3 2.2–5.2
HDL				
Male <40 mg/dl, female <50 mg/dl Male ≥40 mg/dl, female ≥50 mg/dl	3.8 1.8	2.7–5.5 1.0–3.3	5.1 2.4	3.9–6.8 1.5–4.0
LDL				
≥ 100 mg/dl < 100 mg/dl	3.3 1.4	2.4–4.6 0.4–4.5	4.1 2.9	3.1–5.4 1.3–6.4
Hypertriglyceride				
≥ 150 mg/dl < 150 mg/dl	4.2 2.3	2.8–6.2 1.4–3.8	5.6 2.8	4.1–7.6 1.9–4.3
Total	3.0	2.2–4.1	4.0	3.1–5.1

In Tables 2 and 3, in order to know about the dominant risk factors of KD in DM, analysis was performed for respondents of UDDM. Respondents of DDM were excluded to prevent the bias because those DDM might be given intervention by the doctors those influenced risk factors like hypertension, obesity those might be lost.

**Table 2:** Associated risk factors with kidney dysfunction in UDDM

Variables	Significant	OR	95% CI
Age (years)			
20–34		1	
35–54	0.03	9.3	1.23–70.30
Sex			
Male vs female	0.029	2.16	1.08–4.3
Residence			
Rural vs urban	0.168	1.70	0.79–3.62
Education level			
Graduated from primary school or not		1	
Graduated from Junior/ Senior High School/ Academy /University	0.116	1.7 -	0.87–3.2 -
Occupation			
Employee/private business		1	
Jobless		1.32	0.51–3.4
Farmer/Fishermen/Laborers Others	0.341	2.11 2.63	0.82–5.37 0.39–17.46
Economic Status			
High		1	
Middle Low	0.047	2.39 2.72	1.10–5.19 1.12–6.63
Sugary food/drinks			
Not every day	0.01	1	
Every day		2.86	1.21–6.74
Salty food			
Very rarely (< 3×/month) or never		1	
Every day (≥1×/day) Not every day (1–6 x/week)	0.77	1.27 0.98	0.61–2.6 0.46–2.06
Coffee			
Every day		1	
Not every day Very rarely or never	0.239	1.36 1.97	0.54–3.46 0.88–4.40
Smoking			
Never		1	
Every day/any current/former	0.79	1.12	0.46–2.69
Physical activity			
Sufficient		1	
Lacking	0.65	0.82	0.34–1.96
Hipertension			
No Hypertension		1	
Hypertension	0.57	1.21	0.62–2.35
BMI			
Normal		1	
Overweight and Obese Thin	0.77	0.77 0.61	0.35–1.71 0.11–3.31
Obesity			
No central obesity		1	
Central obesity	0.45	0.77	0.39–1.51
Hypercholesterolemia			
Total cholesterol <200 mg/dl		1	
≥ 200 mg/dl	0.286	1.44	0.73–2.82
HDL			
Male ≥40 mg/dl. female ≥50 mg/dl	0.03	1	
Male <40 mg/dl. female <50 mg/dl		2.13	1.05–4.36
LDL			
< 100 mg/dl		1	
≥ 100 mg/dl	0.18	2.34	0.66–8.22
Hypertriglyceridemia			
< 150 mg/dl	0.08	1	
≥ 150 mg/dl		1.80	0.92–3.52

**Table 3:** Associated dominant risk factors with kidney dysfunctions in UDDM (multivariate)

Variables	Significant	OR	95% CI
Age (years)			
20–34		1	
35–54	0.03	9.14	1.19–70.17
Sex			
Male vs female	0.006	2.47	1.29–4.81
Economic Status			
Quintile 4 & 5 (high)		1	
Quintile 3 (middle) Quintile 1 & 2 (low)	0.03	2.70 2.82	1.26–5.81 1.14–6.99
Sugary food/drinks			
Not every day		1	
Every day	0.03	2.61	1.11–6.09
HDL			
Male ≥40 mg/dl, female ≥50 mg/dl		1	
Male <40 mg/dl, female <50 mg/dl	0.011	2.54	1.24–5.19

Table 2 showed that 35–54 year olds had the risk of KD in UDDM 9.3 times higher (CI 1.23–70.3, p 0.03) compared to 20–34 year olds. Male had the risk of KD 2.16 x (CI 1.08–4.3, p 0.029) compared to female. Those with low and middle economic status had higher risk of KD 2.72× (1.12–6.63, p 0.047) and 2.39× (1.10–5.19, p 0.047) than the high economic. People with UDDM who liked to consume sugary food/drinks every day had the risk of KD 2.86 x (CI 1.21–6.74, p 0.01) compared to who did not consume sugary food/drinks every day. People with low HDL had the risk of KD 2.13× (CI 1.05–4.36, p 0.03) compared to those with normal HDL.

Table 3 showed the significant associated dominant risk factors with KD in UDDM after adjustment by the multivariate analysis: the older age (OR 9.14, 95% CI 1.19–70.17, p 0.03), male (OR 2.47, 95% CI 1.29–4.81, p 0.006), low and middle economic (OR 2.7, 95% CI 1.26–5.81, p 0.03) and (OR 2.82, 95% CI 1.14–6.99, p 0.03), eat/drink sugary food/drinks every day (OR 2.61, 95% CI 1.11–6.09, p 0.03), and low HDL (OR 2.54, 95% CI 1.24–5.19, p 0.011).

## Discussion

The prevalence of DM among productive age Indonesian based on *Riskesdas* 2013 was 6.5%, while the prevalence of DDM was only 1.8%. Mihardja L. et al. found that the prevalence of DM among productive age in urban based on *Riskesdas* 2007 was 4.6% and the prevalence of DDM was 1.1% [22]. So, the prevalence of DM was increasing, and many people didn’t know they were suffering from DM. The prevalence of kidney dysfunction among productive age Indonesian was 1.7%, and diagnosed KD was only 0.3%. Stakeholders need to promote early detection of diabetes mellitus and kidney dysfunction in community. We found different result with Prodjosudjadi W. et al. (2009), who found the prevalence of CKD was 12.5% (using CG) or 8.6% (MDRD) [7]. The difference occurred because the study’s sample included younger age than the sample in Prodjosudjadi’s study (20–54 vs 18–70 y.o), in addition to subjects living in urban rural area vs subjects living in 4 capitals. Moreover, we analyzed KD using CKD-EPI equation [23]. Steven LA. et al. (2011) with 116,321 kidney early evaluation program (KEEP) participants data found that the prevalence of eGFR <60 mL/min/1.73m^2^ in 18 y.o or more was 14,3% by using eGFR CKD-EPI equations [24]. Coll de Tuero et al. (2012) found that prevalence of renal impairment (GFR < 60 ml/min/1.73m^2^) in a random sample in primary care of 2642 patients with older age of a Mediterranean area was 22.9% (MDRD) [25]. We got a lower prevalence because the research was done in the community, not in health facilities and the subjects were of younger age.

The prevalence of KD in DM was 4% and diagnosed KD in DM was only 0.6% in community. Van der Meer et al. (2010) found more than one-quarter of diabetic patients, aged ≥25, had CKD in Dutch primary health care [26]. Janmohamed MN. et al. found high prevalence of CKD (24.7%, CG equation) among adult diabetic outpatients at a clinic in Tanzania, and they were usually undiagnosed [27]. They found high prevalence because CKD was determined including those with normal glomerular filtration rate as long as there was microalbuminuria.

The prevalence of KD in UDDM was higher in association with factors such as age, male, rural, low and middle economic, sugary food/drinks, salty food, coffee, smoking, hypertension, hypercholesterolemia, low HDL, high LDL, and hypertriglyceridemia (Table 1). It was statistically significant only in the older age, male, low and middle economic, eat/drink sugary food/drinks every day and low HDL in both bivariate and multivariate regression analysis. Van der Meer V et al. (2010) found increased age had the strongest association with decreased eGFR (OR 2.73; CI 95% 2.02–3.70) [26]. Nitta K et al. (2013) also found the same thing. The kidneys were affected by the aging process, which caused numerous effects on the renal system [28]. Anupama (2014) found that an increase of 1 year in age carried 4% chance of getting CKD (CI 95%: 2.9–5.2%) [29]. KD in DM had been found at young ages of 20–34 years. Tong A. et al. (2013) reported young adults with CKD had a low quality of life value. It is important to do early detection in addition to creatinine serum test, as well as examination of urine protein which is an early and sensitive marker of kidney damage [30].

In this study, male had a risk 2.47× higher risk of KD than female (CI 95%: 1.29–4.81. p 0.006) compared to female. Anupama et al. also found that males were more likely to get CKD than females in a rural community in South India [29]. However, Coll de Tuero found significant association between the female gender (OR 2.20; CI 95% 1.86–2.59) and renal impairment [25]. Rodriqest Poncelas A. et al. (2010) in primary care consulting in Spain using a national cross-sectional study found these variables: age, sex (women), systolic arterial blood pressure, and previous history of cardiovascular disease were significantly associated with CKD in DM type 2. Moreover, he found there was an association of occult CKD with female sex (OR 2.7; CI 95% 1.83–3.99) [31]. The sex difference may be caused by different lifestyles; this requires further research.

Consuming sugary meals everyday had risks 2.61× higher (CI 95%; 1.11–6.09, p 0.03) compared to 1-6×/week or less. Johnson et all (2007) in review found that in developing countries increased rates of sugar consumption also increased cardiorenal disease [32]. We found the prevalence of KD in UDDM in people who liked to eat salty food didn’t differ significantly by bivariate analysis. However, consumption of salty food must be controlled. Lambers HJ et al. provided an assessment in an article review saying that high-salt intake induced hyper filtration that could cause renal damage [33]. Prevalence of KD in UDDM was lower in people who liked to drink coffee every day than those who did not consume coffee every day/never (Table 1). Kim et all (2008), in the Fourth Korea National Health and Nutrition Examination Surveys, showed that coffee consumption was significantly associated with a decreased risk of renal impairment. Coffee contains many antioxidants like caffeic acid, hydroxyhydroquinone, and chlorogenic acid those might protect the glomerular endothelium from oxidative stress [34].

Prevalence of KD in UDDM was lower among those who lack physical activities compared to those in sufficient-physical-activity group (univariate). Usually, sufficient physical activities give the benefit to prevent non-communicable diseases. Nunan et al., using a systematic review, found that exercise-only interventions were no better than usual care alone or diet interventions. To assess the success of physical activity, we needed to know the content of physical activity interventions (type, intensity, and duration), behavioral aspects, and measurement of physical activity levels [35]. The other possibility may be the subjects of sufficient-physical activity had large muscle volume and increased serum creatinine level which lead to lower GFR. On the other hand, subjects of slight KD zone defined as normal in lack of physical activity because of relatively lower serum creatinine level. Banfi G reported the concentrations of serum creatinine in athletes were higher than those found in sedentary people and a positive correlation occurred between BMI and serum creatinine [36].

Prevalence of KD in hypertension group (3.6%) was higher than in no hypertension group (2.6%), but not significanly different. Rodriqest Poncelas A. et al. found that blood pressure was associated with CKD in diabetic patients if the blood pressure was higher than 150/100 mm/Hg [31]. Joint National Committee (JNC) 8 and KDIGO guidelines has recommended that in order to slow CKD progression and to reduce CVD mortality, the blood pressure level must be below 140/90 mmHg [37]. Nugroho P. et al. [38] found that hypertension is associated with kidney dysfunction in Indonesia population.

BMI in this study showed no association with KD. Lu JL, et al. (2015) found BMI of 30 kg/m^2^ or more was associated with rapid loss of kidney functions, but this association was accentuated in older patients (not young patients like in this study) and BMI 25- < 30 was associated with the best clinical outcomes [39].

In this study people with dyslipidemia had the risk associated with KD in DM; low HDL was one of the dominant risk factors**.** Tannock L found CKD was associated with a dyslipidemia, comprised of elevated triglycerides and low HDL-cholesterol [39]. Dyslipidemia was known as a risk factor for cardiovascular disease (CVD). Most patients with CKD died as a result of cardiovascular complications rather than end-stage renal disease [40]. Early detection of kidney disease’s risk factors in patients with DM and education are important for preventing the onset of kidney disease and complications of cardiovascular disease [5, 41].

The limitations of this study was due to a cross sectional study which didn’t explain about causal relationships. A cohort study is needed for further study. The other limitation the prevalence was defined by only serum creatinine. Consequently, the prevalence in this study showed lower value than other reports including microalbuminuria with normal glomerular filtration.

## Conclusion

The prevalence of KD in DM among productive age Indonesian was 4.0% and only 0.6% had been diagnosed. Early detection of KD in DM is needed in order to slow progression and to prevent complications. The dominant risk factors associated with KD in DM were age, male, economic status, sugary food/drinks, and low HDL. Control of risk factors in DM related to lifestyle should be done in order to prevent diabetic kidney disease. National health promotion activities in Indonesia must be increased and intensive screening for diabetes should be stress in publich health care to reduce the burden of CKD.

## Abbreviations

*BMI*: body mass index
*DDM*: diagnosed diabetes mellitus (DDM)
*DM*: diabetes mellitus
*KD*: kidney dysfunction
*UDDM*: undiagnosed diabetes mellitus
